# Systematic review on factors associated with depression among mothers of children with cancer

**DOI:** 10.1371/journal.pone.0285366

**Published:** 2023-08-24

**Authors:** Wan Syahirah Wan Ghazali, Halimatus Sakdiah Minhat, Nor Afiah Mohd Zulkefli, Norliza Ahmad, Fatin Ismail, Dina Nurfarahin Mashudi, Muhammad Ikhwan Mud Shukri, Chandramalar Kanthavelu

**Affiliations:** Universiti Putra Malaysia, Serdang, Selangor, Malaysia; Rutland Regional Medical Center, UNITED STATES

## Abstract

**Background:**

Despite evidence of depression among mothers of children with cancer, there appears to be a lack of studies or concern regarding factors associated with depression among these mothers.

**Objective:**

To review the factors associated with depression among mothers of children with cancer.

**Method:**

Pubmed, Medline, Cochrane, CINAHL, Psychology, and Behavioural Sciences Collection, and Academic Search Complete were searched according to Preferred Reporting Items for Systematic Reviews and Meta-Analyses (PRISMA) guidelines to identify studies published between 2010 to 2022 on the associated risk factors of depression among mothers of children with cancer. The keywords used included mothers OR maternal’ AND ‘Child*’ AND ‘cancer OR tumo*r OR neoplasm’ AND ‘factors OR facilitators AND barriers OR predictors OR determinants AND ‘depression’. Selected studies were evaluated by quality assessment.

**Result:**

Five articles fulfilled the eligibility criteria. The factors associated with depression among mothers of children were socio-demographic risk factors (marital status, education level, annual income, child cancer diagnosis), and stress factors (caregiving stress, cancer-related stress, general stress). There were other factors associated with depression that act as mediators along the process which were emotion-focused coping and perceived social support.

**Conclusion:**

Besides the commonly reported socio-demographic risk factors (marital status, education level, and annual income), other factors include stress factors (caregiving stress, cancer-related stress, and general stress). Furthermore, emotion-focused coping and perceived social support act as mediators along the process. More studies are warranted to explore depression among these mothers to ensure the most appropriate and effective preventive measures.

## Introduction

Childhood cancer is the leading cause of death among children and adolescents globally. The World Health Organisation (WHO) estimated that 400,000 children and adolescents develop cancer each year [1]. For the past decade, there are advancements in the diagnosis and treatment of childhood cancer. Regardless, childhood cancers remained the most influential childhood chronic diseases that contribute to whole new challenges to the family [1,2].

Learning the child has a life-threatening illness is a traumatic event for the parents. The additional stressors of intensive treatment regimes, frequent hospital visits, marital challenges, financial difficulties, balancing responsibilities with other family members, and the constant fear of relapse or death may compound the mental health of the parents. One of the most common illnesses suffered by parents is depression. A meta-analysis among parents of children with cancer showed that the pool prevalence of depression is 28%, followed by Post-Traumatic Stress Disorder (PTSD) at 26%, and anxiety at 21% [2].

Among parents with depression, studies have shown that mothers are more vulnerable to depression than fathers [2–7]. Depression was reported in 77.2% of mothers and 57.1% of fathers of children with cancer in Basrah, Iraq [4]. In Turkey, 36.4% of the mothers and 25% of the fathers had severe depression, 18.2% of mothers and 4.5% of fathers had moderate depression [3]. Furthermore, mothers are more involved in caregiver tasks than fathers [2,5]. In a meta-analysis among 9262 parents of children with cancer, 6606 (71%) were mothers and only 2685 (29%) were fathers [2]. Similarly, in a systematic review on parental health and well-being involving 3441 caretakers of children with cancers, 2261 (66%) of the caregivers were mothers, 1099 (32%) were fathers and 80 (2%) were others [5]. Therefore, in view of the high prevalence of depression among mothers who play the role as the main caretakers, this systematic review will be focussing on maternal depression rather than depression among the parents.

There are several explanations for the higher depression level among mothers. An explorative study by Young et al. [2002] suggested that mothers experience the most consequences when family members suffered from chronic illnesses. In the relationship of mothers and children with cancer, the biographical disruption began the moment mothers noticed changes in their child, intensified with the diagnosis which subsequently altered the mother’s self-efficacy and social identity [6]. Biographical disruption is a concept introduced by British Sociologist, Michael Bury in 1982 to describe the influence of chronic illness on threatening the individual’s self-identity that breaks the social and cultural experience of that individual [7]. Among mothers, the sudden, unexpected event of having a child with chronic illness unexpectedly change the mother’s direction and plans in life. It brought a new set of responsibilities to the mothers, including the obligation of ‘proximity’ to the sick child to provide ‘comfort’ and ‘keep watch’ all the time [4].

In addition, the caregiving role create an intense emotional interdependence between the mothers and the sick child, in a way mothers ought to share and carry the burden of their child; to the extent of wishing to swap places with the child [4]. Dealing with all these obligations compromised the mother’s role to other children and relationship with other family members [4]. Even after the completion of cancer treatment, the fear of uncertainty and the possibility of recurrence will always be a major concern [8]. These stressors act as risk factors for increasing the risk of depression among the mothers.

In term of roles and responsibilities, mothers play more roles of nurturing and caring for the child whereas fathers take the role of financial stability [9]. In South Korea for example, despite the rapid industrialization since 1960, the women’s responsibilities are primarily nurturing and domestic task which are related traditionally to the strong patriarchal society [9]. Similarly, in the United States, a large population survey was conducted to highlight the distinct roles play by fathers and mothers at home. The survey used the data from the American Time Use Survey (ATUS) involving 137,000 respondents from 2003–2012 and the 2012 American Community Survey involving 1% sample of the US adult population. The study concluded that mothers spend more time each day accommodating to the needs of the young child compared to fathers [10]. In addition, in occupational field, more women were involved in nurturing profession compared to protective professions [10]. Different occupations were categorised either into nurturing professions (childcare, nurse, elementary school teacher, or home care aide) or protective professions (security guard, firefighter, police officer, or armed forces). The survey found that less women were involved in the protective professions in which 95% of firefighters, 88% of soldiers, 86% of police officers and 79% of security guards were male. In contrast, in the nurturing professions, only 2.3% of informal day care provider, 2.1% of kindergarten teacher and 5.8% of workers in the childcare industry were male [10]. These findings showed the different roles between fathers and mothers, in which mothers were more involved in nurturing role whereas fathers were more involved in protective roles [9].

Apart from that, the impact of maternal depression is not only to the mothers, but to the other family members including the spouse, other children, and the sick child. Their caregiving abilities may be impeded with the presence of depression. In addition, previous literature had shown that highly distressed mothers contribute to highly distressed child [11]. Hence, it is important to recognize and alleviate factors associated with depression among these mothers to improve the whole family survivorship experiences and quality of life following the cancer diagnosis and treatment [11]. The findings on this review will allow relevant authorities to plan and institute policies and preventive measures, thereby enabling mothers of children with cancer to have better mental health, thus, provide better caregiving to their children and families. Therefore, the main objective of this study is to review the factors associated with depression among mothers of children with cancer.

The factors identified were categorised into three domains based on Social Cognitive Therapy (SCT) which consisted of personal, behaviour and environment factors. SCT theory was developed by Bandura in 1989 that explains human behaviour in a three way reciprocal model (personal, behaviour, environment) that continually interact with each other [12]. There were limited studies explained factors associated with depression among mothers based on SCT constructs, however several studies have applied SCT based mental health intervention to the caregivers of chronic illnesses including children with cancer. Hence, SCT is chosen as the theoretical framework for this review to provide better understanding on the development of intervention module to reduce depression among mothers of children with cancer.

## Materials and methods

**Fig 1:**
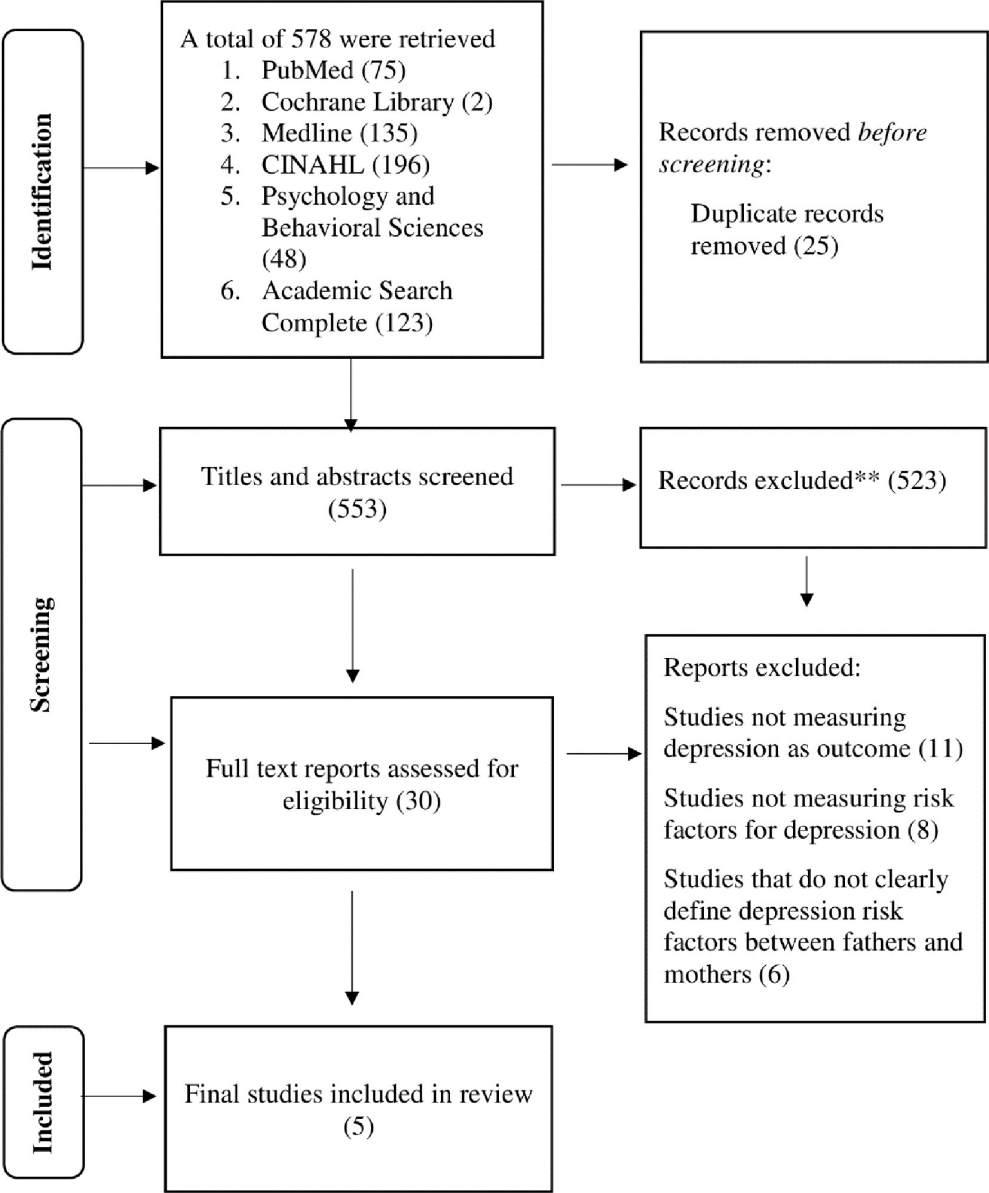
PRISMA flow chart.

A systematic search was performed to identify factors associated with depression among mothers of children with cancer using six electronic databases, including Pubmed, Medline, Cochrane, CINAHL, Psychology and Behavioural Sciences Collection and Academic Search Complete. This systematic review was conducted in line with the Preferred Reporting Items for Systematic Reviews and Meta-Analyses (PRISMA) guidelines **(Fig 1).**

### Eligibility criteria

This review included articles meeting the following criteria: [a] articles with English version; [b] articles with available full text; [c] original research papers, peer-reviewed or published articles; [d] published between 2010 and 2022; [e] articles with the keywords detailed in Table 1. The keywords used were ‘mothers OR maternal’ AND ‘Child*’ AND ‘cancer OR tumo*r OR neoplasm’ AND ‘factors OR facilitators AND barriers OR predictors OR determinants AND ‘depression’. The Mesh term was used for ‘depression’ and ‘tumo*r’ as the terms slightly varied between databases.

**Table 1:** Keywords used for the literature search.

Mothers	Mother* OR maternal
	AND
Factors	Factors OR predictors OR determinant OR ‘facilitators AND barriers’
	AND
Cancer	MeSH [Tumo*r]
	AND
Depression	MeSH [depression]

### Selection of literature (screening and eligibility)

Using the stated keywords, relevant literatures were identified following a comprehensive literature search of six electronic databases. Studies that were not published between January 2010 to December 2022, without available full texts, not in English language were excluded using the filtering tools of the database. All the findings from the literature searches were subsequently transferred into Microsoft Excel. The list of literature which fulfilled the criteria was further screened by two reviewers based on the title, abstract, keywords, and statement in eligibility criteria. Any questionable articles or discrepancies were further discussed with the third reviewer. Studies with irrelevant information, duplicated publications, and review articles were removed.

As shown in Fig 1, out of 578 potential articles, 25 duplicate articles were removed before screening. The remaining 553 articles were screened based on their titles, abstracts, and keywords. This screening process yielded 30 articles which met the eligibility criteria. The relevant data from each article include the authors, year of the study, study population, sample size, methodology or instrument used to assess depression and risk factors. From the remaining articles, a total of 25 articles were further excluded; 11 articles were excluded as depression was not measured as the outcome of the studies, 8 articles did not report risk factors of depression and 6 articles did not clearly define depression risk factors between fathers or mothers. Finally, a total of five studies fulfilled all eligibility criteria for this review.

### Quality assessment of the literature

A quality assessment was carried out on the selected articles using the Crowe Critical Appraisal Tool (CCAT) [13]. The CCAT quality assessment tool consisted of 22 items, divided into eight categories as follows: [a] preliminaries (title, abstract and text), [b] introduction (background and objective), [c] design (research design, exposure or treatment, outcome and measure and bias evaluation), [d] sampling (size and sampling protocol) [e] data collection (collection method and protocol) [f] ethical matters, [g] results (analysis, interpretation, and outcome), [h] discussion. The score for each item ranged from the lowest of 0 to the highest of 5. The overall score of 40 is then converted into percentage whereby the quality of each article was categorised into poor quality (≤50%), acceptable quality (51–74%), high quality (≥75%) [13]. The consensus of quality assessment by two reviewers is shown in **Table 2**.

**Table 2:** Description of the quality markers.

Item	Category	(Howard et al., 2019) [14]	(Bemis et al., 2015) [15]	(Sulkers et al., 2015) [16]	(Barrera et al., 2012) [17]	(Demirtepe-Saygili & Bozo, 2011) [18]
1.	Preliminaries (/5)	3	5	4	4	4
2.	Introduction (/5)	4	5	4	4	5
3.	Design (/5)	4	4	4	4	4
4.	Sampling (/5)	3	3	3	3	3
5.	Data collection (/5)	4	4	3	3	5
6.	Ethical matters (/5)	3	4	4	3	4
7.	Results (/5)	5	5	4	3	5
8.	Discussion (/5)	5	5	3	4	5
9.	Total Score (/40)	31	35	29	28	35
10.	Percentage (%)	78%%	88%	75%	68%	88%

## Results

In the five articles reviewed, two studies were from the United States, one study from Canada, one study from the Netherlands, and one study from Turkey. Three articles studied mothers of children with cancer, one article studied both fathers and mothers, and one article studied mothers and children with cancer. From the two studies that involved both ‘fathers and mothers’, and ‘mothers and children’, the findings of factors associated with depression were further divided into maternal, paternal or child factors. Only the maternal factors from the findings were subsequently included in this review.

The overall sample size in this review was 719 mothers with a minimum sample size of 69 and a maximum of 327. As for the age of the children, two articles mentioned children aged below 18 years old, two articles mentioned children aged from 5 to 17 years old and one article mentioned children more than 3 years old. Overall, all the articles studied mothers of children aged less than 18. In term of the study design, three out of five studies used a prospective longitudinal study design in which the duration of follow-up ranged from diagnosis or from relapse until five years post-diagnosis. Another two studies used a cross-sectional study design. One study used Pearlin Caretaker Model as the theoretical framework of the study. The CCAT quality assessment scores of these 5 articles ranged from 68% to 88% indicating all the chosen articles were either acceptable (51–74%) or high quality (>75%). The risk factors of five selected studies were summarised in Table 3.

**Table 3:** 

Author & Year	Study Population, Study location & Sample Size (N)	Study design	Methodology/ Instrument	Risk Factors		
				Personal	Behaviour	Environment
(H. Sharp et. al., 2019) [14]	Mothers of children with cancers, United States, (327) Children aged 5–17 years	Prospective Longitudinal study (near the time of disgnosis/ relapse, at 1-, 3- and 5-years post diagnosis.	Beck Depression Index (BDI -II)		Primary and secondary control coping were predictive of mothers’ trajectory membership. Primary control coping was more likely to be assigned to low trajectory depression as compared to high depression (OR = 1.99, p = 0.008) Primary control coping was more likely to be assigned to low trajectory depression as compared to moderate depression (OR = 1.64, p = .024) Secondary control coping was more likely to be assigned to low trajectory depression as compared to high depression (OR = 1.81, p = 0.001) Secondary control coping was more likely to be assigned to low trajectory depression as compared to moderate depression (OR = 1.38, p = .013) More seco control coping also predicted that mothers were more likely to be assigned to the moderate depression trajectory as compared to the high depression trajectory (OR = 1.31, p = .035).	
(Bemis et. al., 2015) [15]	Mothers of children with cancers (N = 138), Children aged 10–17 years with cancers (N = 151), United States	Cross sectional study	Beck Depression Index (BDI -II)	**Bivariate Analysis** Single mothers (p<0.05), annual family income (p<0.01), mother’s educational level (p<0.001) **Multivariate analysis** General Stress (p<0.001) Cancer related stress (p<0.001)		
(Sulkers et al., 2015) [16]	Mothers of children with cancer (N = 95), Netherland Children aged below 18 years	Longitudinal study, (at diagnosis, 3, 6 and 12 months thereafter)	Beck Depression Inventory (BDI;	High caregiving stress was associated with more depressive symptoms (OR = 7.25, 95% CI 6.39, 8.12, p<001) Diagnosis of child cancer: Brain tumour (Ref) Haematological tumour (OR = - 4.56. 95% CI -7.30, -1.83, p = 0.01) Solid tumour (OR = -4.07. 95% CI -6.96, -1.17, p = 0.07)		
(Barrera et al., 2012) [17]	111 parents of children with cancer, (69 mothers and 42 fathers), Canada Children aged more than 3 years old who were eligible for transplant	Longitudinal study (before Stem Cell Transplant (SCT), 1- and 2-years post SCT), Canada	Beck Depression Inventory (BDI)	Mothers’ depression score pre SCT significantly predicted mothers’ depression a 2 years post diagnosis (p<0.0001) Child diagnosis significantly predicted mothers’ depression a 2 years post diagnosis (p = 0.013) Pairwise analysis showed higher depressive scores for mothers whose children were diagnosed with neuroblastoma compared with mothers whose children were diagnosed with blood disorders (p = 0.0126)	Mixed linear model A significant positive association between maternal depression and the child behaviour scores (P = 0.0496)	
(Demirtepe-Saygili & Bozo, 2011) [18]	90 mothers of children with Leukemia, Turkey Children aged below 18 years	Cross sectional study	Beck Depression Inventory	Factors that were significantly associated with the level of depressive symptoms were age (β = -0.19, p<0.05) and education level (β = 0.64, p<0.001 **Predictors** Basic needs (β = -0.37, p<0.001) Role strain (β = -0.34, p< 0.001)	Problem-focussed coping was not a significant predictor of depressive symptoms (β = -0.01, p = 0.88). Emotion-focussed coping significantly predicted depressive symptoms while controlling for basic needs (β = 0.30, p<0.001). Emotion-focussed coping was a significant predictor of depressive symptoms while controlling for role strain (β = 0.31, p<0.001). Emotion-focussed coping mediated the relationship between basic needs/ role strain and depressive symptoms.	Perceived social support significantly predicted depressive symptoms, while controlling for basic needs (β = 0.24, p<0.05). Perceived social support was a significant predictor of depressive symptoms, while controlling for role strain (β = 0.21, p<0.05). Perceived social support mediated the relationship between basic needs/ role strain and depressive symptoms

### Factors associated with depression

**Fig 2:**
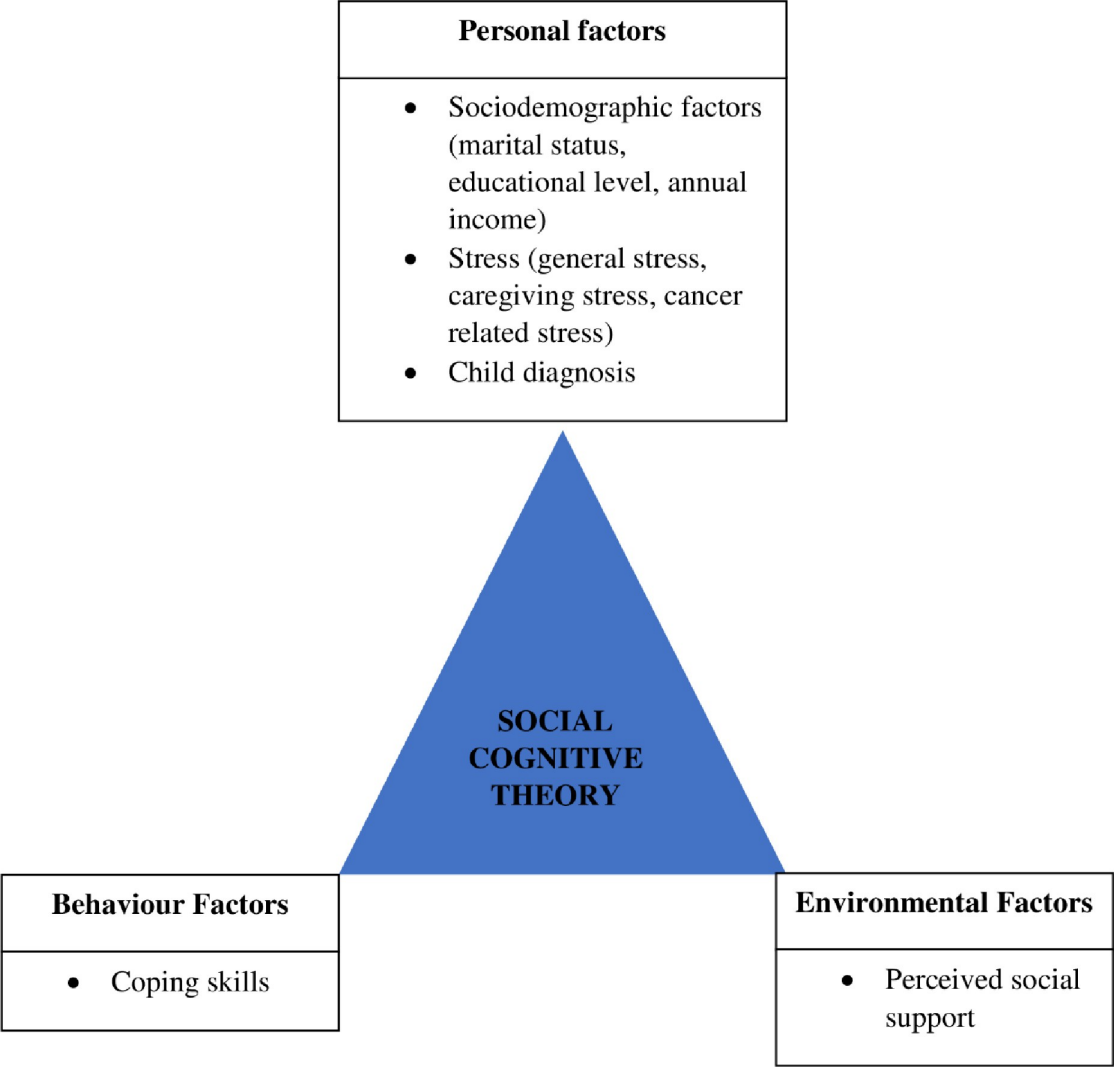
Factors associated with depression among mothers of children with cancer based on Social Cognitive Theory.

Factors associated with depression among mothers of children with cancer identified from the review of the articles were further categorised into personal, behaviour and environment domain based on Social Cognitive Theory as shown in **Fig 2.**

In the personal factors, single mothers [15], lower educational level [15,18] and lower annual family income [15] were factors associated with depression among these mothers. Contrary, there were different findings on maternal age and risk of depression. One study reported maternal age was not found to be risk factors for depression [15] and another study showed younger maternal age were associated with high risk of depression [18]. Therefore, in view of this contradicting findings, maternal age was excluded as one of the risk factors for depression in this review.

In term of the child diagnosis, mothers whose children were diagnosed with haematological disorder and solid tumour were less likely to have depression compared to mothers with children of brain tumour [15]. Another study reported child diagnosis significantly predicted maternal depression at two years post diagnosis, in which mothers having children diagnosed with neuroblastoma showed higher depressive score compared with mothers whose children were diagnosed with blood disorders [17].

Furthermore, review of these articles found that higher stress was associated with higher depression levels among the mothers [14,16]. Mothers with high caregiving stress [16], cancer related stress [15] and general stress [15] were at risk of developing depression. Apart from that, a longitudinal study was conducted among mothers of children who had Stem Cell Transplant Treatment (SCTT) up to 2 years post SCTT [17]. The findings of the study concluded that maternal depression score pre SCTT predicted the maternal depression score at 2 years post SCTT [17]. Another 5 years follow up longitudinal study reported that on average, maternal depression symptoms reduced over time [14]. However, the severity of depression generally remains the same throughout the follow up. For example, mothers who were categorised as having ‘High Depression Symptoms’ at diagnosis or relapse would remain in the same category after 5 years follow up despite of the significant decreased of the depression symptoms over time [14]. These findings therefore indicate the need for early intervention for mothers experiencing depression or mental health problems at diagnosis [14].

One study showed ‘basic needs’ and ‘role strain’ were predictors of mother’s depression, in which basic needs includes not only the physical needs like sleep and nutrition but also some other needs like expression of feelings, relaxation and personal growth [18], meanwhile role strains include hardships in tasks that need to be completed by the caregiver apart from the caregiving, such as job, economic problems and social life.

In Social Cognitive Theory, maternal coping responses to the child’s cancer were categorised under the behavioural factors of the theory. In a longitudinal study by H. Sharp et al. [2019], coping factors among mothers were categorised into three categories, primary coping which includes active, voluntary efforts to alter the situation or a person’s emotional state (e.g., problem-solving, emotional regulation, emotional expression), secondary control coping refers to efforts to adapt to or fit into present conditions (e.g., positive thinking, cognitive restructuring, acceptance) and disengagement coping refers to voluntarily retreating from addressing or acknowledging stressors (e.g., avoidance, denial,). The study found that mothers with primary and secondary coping were less likely to have depression during the five years following the child cancer’s diagnosis or relapsed [14].

Meanwhile, another study by Demirtepe-Saygili & Bozo [2011] had categorised coping into problem-focused coping and emotion-focused coping. Based on Pearlin’s Caretaker Model as the theoretical framework, the study investigated the role of problem-focused coping and emotion-focused coping to depression among the mothers. The findings of the study showed problem focused coping had negative correlations with depressive symptoms whereas emotion focused coping had positive correlations with depressive symptoms. Mothers who used more problem focused coping had fewer depressive symptoms, however, mothers with more emotion focused coping had more depressive symptoms. The study also found that emotion-focused coping mediated the relationship between the ‘stressors’ and depressive symptoms among the mothers [18]. The stressors identified in this study were the ‘basic needs’ and ‘role strain’ of the mothers [18]. The study proposed that caregivers, who met their basic needs less, were more likely to use emotion-focussed coping and thereby reported more depressive symptoms. Similarly, caregivers who experienced higher role strain, in other words, who were less satisfied with their non-caregiving activities, were more likely to use emotion-focussed coping, which in turn increases the likelihood of caregivers to experience more depressive symptoms [18].

As for the environment factors, Demirtepe-Saygili & Bozo [2011] showed that perceived social support mediated the relationships between basic needs and role strain and depression among the mothers. Caregivers who met their basic needs more, were more likely to report fewer depressive symptoms through perceiving higher social support. Similarly, caregivers who experienced higher role strain reported less perceived social support, and thus, experienced more depressive symptoms [18].

## Discussion

The results of this review demonstrate the apparently limited number of studies specifically focussing on depression among mothers of children with cancers. Among those reviewed, three studies used mothers as the study populations [13,18,19]. One study used mothers and the child with cancers as the study population [15] and another study used both fathers and mothers as the study populations [17]. There was harmonious use of the Beck Depression Index (BDI) as the main instrument for determining depression in four studies. Only one study used the Centre for Epidemiologic Studies Depression Scale (CESD) as the base instrument for depression [16].

In term of risk factors, the studies in this review revealed homogenous risk factors for depression among mothers of children with cancers. As for the sociodemographic risk factors, two studies from this review found that the mother’s educational level was negatively associated with the mother’s depression [15,18]. Mothers with higher educational level were more involved with the child’s treatment, hence more understanding of the child’s condition and treatment plan. A previous study among parents of children with cancers in Malaysia found that highly educated parents were more willing to seek information from health care providers [11]. The involvement and participation of mothers in the childcare may increase the mother’s self-esteem and at the same time reduce mothers’ depressive levels [11].

Apart from that, one study from this review identified annual family income was negatively associated with depression among these mothers This finding was in line with findings from a study conducted among parents with childhood cancer in Lebanon in which family financial problem was one of the predictors of psychological distress among the mothers [20]. Even though this study focused on factors associated with psychological distress and not merely depression, psychological distress may indicate the early symptoms of other mental illnesses including depression, anxiety and stress [21]. Ironically, in a study conducted among parents of children with Leukaemia in Malaysia, family income was not statistically associated with parental distress [22]. The differences might be because the cost of chemotherapeutic treatment in Malaysia was heavily supported by the Malaysian Government [22].

In term of age, there were contradictory findings from 2 studies [15,18]. Only one study in this review identified the association between age and the mother’s depressive symptoms [18]. The younger the age of the mothers, the higher the association with the mother’s depressive symptoms. Similar findings were reported on maternal stress among mothers of children with cancers in Paris, in which mothers with younger age had a higher maternal stress as compared to mothers with older age [23]. Looking at the age factor, older mothers can be assumed to have better knowledge about childhood cancer as well as having a better experience in coping with eventful life stressors such as their child’s illnesses. In addition, ‘older mothers’ might be able to benefit more from the social support network as compared to the ‘younger mothers’ [20].

In addition, mothers with high stress level were more vulnerable to have depression. These stressors include caregiving stress [16], cancer related stress [15] and general stress [15]. This is particularly true in paediatric oncology care, because caregivers are usually the parents, who suffer from both parenting and caregiving stress [24].

In term of coping skills, there were various approach used by different studies to assess coping particularly among mothers of children with cancer. The findings from this review categorised coping into problem-focused coping and emotion focused coping [18] or primary, secondary, and disengagement coping [14]. Among Korean mothers of children with cancer, coping skills were categorised coping as Coping Skills 1 (Maintaining Family Integration and an Optimistic Outlook for the Situation), Coping Skills II (Seeking Social Support) and Coping Skills III (Seeking Information) [9]. The variation in reported coping skills among mothers is likely to be explained by variation of tools used to explain coping skills. This heterogeneity in results has led to inconclusive and subjective findings, which prevent the development and resourcing of target intervention among the mothers.

This review highlight the role of perceived social supports as the predictor and mediator for depression among the mothers [18]. This is supported by the Pearlin’s Caretaker Model which regarded social supports as one of the mediators to the stress process [25]. Furthermore, Harper et al. (2016) showed that in parents of children with cancer, the higher the satisfaction of the social supports received, the lower the parental distress [26]. Among mothers of children with cancer in Ankara, only 73.9% claimed they received the required social support when 100% of them claimed the need of social support [27] In a population survey conducted to assess the needs of parents of a child with cancer between 2015 and 2020, 800 caregivers were included in the study [28]. The findings of the study showed that 31% (N = 211) of the female caregivers and 35% (N = 42) of male caregivers regarded the support needed in dealing with depression as moderate and high needs. In terms of psychological support from support group, 20% (N = 136) of female and 15% (N = 18) of male caregivers perceived the needs as moderate and high [28]. Therefore, parent-oriented care particularly targeting mothers of children with cancer should be included in the paediatric oncology department, aiming to understand the unmet supports and unmet needs of these parents, at the same time providing the appropriate intervention to boost the mental health of the mothers as the caregiver.

There are a few limitations of this review. Overall, there appears to be lack of a general review of factors associated with depression among mothers of children in cancer. Studies on depression among caretakers of children with cancers generally were conducted amongst the parents of the cancer child without focussing on the mothers alone. Previous literatures have shown that mothers experiencing higher depression level as compared to fathers. Therefore, depression among mothers warrant for further elaboration and studies to be conducted.

In term of the methodology, the review did not include studies done prior to 2010. Additionally, certain keywords such as ‘Parent*’, ‘Caregiver*’or ‘Caretaker*’ were not included in the search strategy. Thus, it is possible that significant risk factors has been missed and not included in the conclusion of this review.

Apart from that, other limitations of the study were the wide range of mental health term used as the main indicators of the study. Several studies used the term ‘psychological distress’ or ‘wellbeing’ when the study outcome was ‘depression’. Hence, there were possibilities these */studies might not be included in the reviews. Furthermore, there were other risk factors of maternal depression that were not mentioned in any study in this review. This includes a personal history of depression, and a history of illicit drug and alcohol use. Therefore, this review may represent some reporting bias given mothers who were experiencing depression were less likely to participate in the study.

Besides the limitations already acknowledged, the type of cancer in this review varies between studies with only one study focussing on children with Leukaemia. Therefore, this review is unable to conclude any relationship between the type or prognosis of cancer and the severity of the symptoms that may exacerbate depression among the mothers.

Finally, the studies in this review were generally among mothers in high-income countries which were United States, Canada and The Netherlands. Only one study was conducted in Turkey, a middle eastern country. None of the study was conducted in the lower income countries. Hence the findings of this review cannot be generalised to other countries with different socioeconomic and cultural background.

### Conclusions and recommendations

In conclusion, despite the lack of study on depression among mothers of cancer, there is cause for concern as depression among mothers have a great negative impact to the mothers, family members and the sick child. Besides the commonly reported socio-demographic risk factors (age, marital status, education level, and annual income), stress factors (caregiving stress, cancer related stress, general stress), and other risk factors such as child behaviour, there were other factors associated with depression which act as mediators along the process (emotion-focussed coping, perceived social support).

By understanding depression and associated factors among this population, it provides insight and opportunity for relevant authorities and stakeholders to address and mitigate depression among mothers of children with cancer through policy planning and targeted planned intervention. However, with the limited number of studies in this review, more studies are warranted to explore depression among this population to ensure the most appropriate and effective preventive measures.

## Supporting information

S1 Checklist PRISMA 2020 checklist. (DOCX)
